# The living heart: Climate gradients predict desert mountain endemism

**DOI:** 10.1002/ece3.7333

**Published:** 2021-03-15

**Authors:** Peter J. McDonald, Peter Jobson, Frank Köhler, Catherine E. M. Nano, Paul M. Oliver

**Affiliations:** ^1^ Secretariat of the Pacific Regional Environment Program Apia Samoa; ^2^ Department of Environment and Natural Resources Northern Territory Herbarium Alice Springs NT Australia; ^3^ Australian Museum Sydney NSW Australia; ^4^ Environmental Futures Research Institute and School of Environment and Science Griffith University Nathan Qld Australia; ^5^ Biodiversity and Geosciences Program Queensland Museum South Brisbane Qld Australia

**Keywords:** climate change, conservation planning, diversity, land snails, plants, refuge, refugia, vertebrates

## Abstract

Mountain regions are centers of biodiversity endemism at a global scale but the role of arid‐zone mountain ranges in shaping biodiversity patterns is poorly understood. Focusing on three guilds of taxa from a desert upland refugium in Australia, we sought to determine: (a) the relative extent to which climate, terrain or geological substrate predict endemism, and (b) whether patterns of endemism are complimentary across broad taxonomic guilds. We mapped regional endemism for plants, land snails, and vertebrates using combined Species Distribution Models (SDMs) for all endemic taxa (*n* = 82). We then modelled predictors of endemism using Generalised Additive Models (GAMs) and geology, terrain, and climate variables. We tested for the presence of inter‐ and intraguild hotspots of endemism. Many individual plant and land snail taxa were tightly linked with geology, corresponding to small distributions. Conversely, most vertebrate taxa were not constrained to specific geological substrates and occurred over larger areas. However, across all three guilds climate was the strongest predictor of regional endemism, particularly for plants wherein discrete hotspots of endemism were buffered from extreme summer temperatures. Land snail and vertebrate endemism peaked in areas with highest precipitation in the driest times of the year. Hotspots of endemism within each guild poorly predicted endemism in other guilds. We found an overarching signal that climatic gradients play a dominant role in the persistence of endemic taxa in an arid‐zone mountain range system. An association with higher rainfall and cooler temperatures indicates that continuing trends toward hotter and drier climates may lead to range contractions in this, and potentially other, arid‐zone mountain biotas. Contrasting patterns of endemism across guilds highlight the need to couple comprehensive regional planning for the protection of climate refugia, with targeted management of more localized and habitat specialist taxa.

## Graphical abstract

We found an overarching signal that, even in a low elevation and relatively low rainfall arid zone refugia, climatic gradients still play a dominant role in the persistence of endemic taxa. Nonetheless individual taxa also show contrasting patterns at local scales, highlighting the need for both conservation strategies targeting at‐risk taxa and comprehensive regional planning for the protection of critical climate refugia.

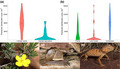

## 1 INTRODUCTION

Mountain regions are global centers of species richness and endemism, particularly at tropical latitudes (Rahbek et al. 2019a). Hyperdiverse tropical mountains are characterized by extreme variation in climate over relatively small spatial scales, likely to be an important factor in promoting and maintaining endemism through climatic change (Rahbek et al. 2019a; Steinbauer et al., 2016). In comparison with lower latitudes, mountainous regions in arctic and temperate zones support relatively fewer species and their biotas are often less distinct from neighboring lowlands (Rahbek et al. 2019a). Patterns of endemism in arid mountain regions are less understood, though there is an emerging recognition that extensive areas of complex arid upland terrain can function as important centers of persistence and diversification, and harbor diverse and endemic biotas (Ashman et al., 2018; Brito et al., 2014; Garcia‐Porta et al., 2017; Pepper et al., 2013).

Two broad and nonmutually exclusive hypotheses to explain the persistence of biota in arid mountain refugia are: (a) upland areas with cooler and wetter climates support species that have been extirpated from nearby lowlands by past shifts toward hotter and drier conditions (i.e., climate relicts), and (b) species are associated with specialized habitats or substrates (i.e., habitat specialists), including those that provide refugia (Couper & Hoskin, 2008; Cox and Moore 2005). The climate relicts hypothesis is based on the understanding that climate is a dominant force shaping species distributions (Cox et al., 2016; Woodward, 1987), with geographically widespread species contracting to upland refugia during periods of aridification or warming (Byrne et al., 2008; Rahbek et al. 2019b). The habitat specialist hypothesis is based on the observation that many species are not at equilibrium with climate and thus occur only in a subset of their fundamental niche (Araújo & Pearson, 2005; Hutchinson, 1957; Pearson & Dawson, 2003). For these habitat specialist species, resilience to climatic change may be achieved through plastic responses and genetic adaptation (e.g., plants; Corlett & Tomlinson, 2020), use of climate‐buffered microhabitats (e.g., burrowing lizards; Moore et al., 2018), and the use of torpor (e.g., dasyurid marsupials; Warnecke et al., 2008) or aestivation (e.g., land snails; Solem, 1993). If both broad climate and local habitat are important in the accumulation of endemism in arid mountain ranges, predictors of endemism could be expected to differ across guilds of biota with different life histories, ecologies and dispersal abilities.

Understanding the relative roles of broader climatic limitation versus habitat specialization in the accumulation of arid mountain biotas is of strong relevance for predicting the ongoing persistence of endemic species and populations. Species for which distributional limits are mostly shaped by broader climate (hereafter termed climate relicts) may be particularly sensitive to climate change, responding for example through upward shifts in elevation (Lenoir et al. 2008; Moritz et al., 2008). For climate relict species that may occur widely across upland areas, identifying and protecting regional‐scale climatic refugia is a key conservation priority (Cañadas et al., 2014). Conversely, while habitat specialists may be somewhat resilient to or buffered from climate change (Corlett & Tomlinson, 2020; Moore et al., 2018; Sánchez et al., 2017), their ecological specialization frequently translates to restricted geographic range sizes and thus increased vulnerability to localized stochastic and disturbance events (Böhm et al., 2016; Purvis et al., 2000; Slatyer et al., 2013). For some of these specialist species, localized management of key threats may be particularly valuable for preventing extinctions. Understanding the predictors of endemism and how much these overlap between biotic guilds is thus of critical importance in identifying and protecting key refugia and in the spatial prioritization of threat management.

The MacDonnell Ranges is an upland region (315–1531 m. a.s.l.) in the middle of the vast Australian Arid Zone (AAZ). Unusually, the MacDonnell Ranges uplifted in the center of a stable continent (~300–450 million years ago (Ma); Shaw et al., 1991), resulting in a region of complex terrain and diverse geologies far from ameliorating coastal influences and surrounded by flat sand deserts. The higher elevations of these ranges are characterized by higher rainfall and cooler temperatures than the surrounding arid plains. The region is established as a center of land snail endemism and plant species richness (Crisp et al., 2001; Slatyer et al., 2007) and phylogenetic studies demonstrate that that the ranges have functioned as a refugium for plants, aquatic invertebrates, land snails, and vertebrates through periods of aridification as early as the mid‐Miocene, and continuing through the Plio‐Pleistocene (Ashman et al., 2018; Cardillo et al., 2017; Christidis et al., 2010; Criscione & Köhler, 2016; Ingham et al., 2013; Oliver et al., 2010, 2014; Oliver & McDonald, 2016; Pepper et al., 2013; Razeng et al., 2017). Given that the MacDonnell Ranges have supported a suite of endemic taxa through major climatic change, it could be expected that climatic gradients will be important predictors of present‐day endemism (i.e., endemics are dominated by climate relicts limited to cooler and wetter regions). However, the low elevation of the MacDonnell Ranges raise the possibility that microhabitats and geological variations, could be more important drivers of persistence and endemism in this system (i.e., endemics are dominated by habitat specialists).

Here, using Australia's MacDonnell Ranges as a focal region, we explore the predictors of endemism in a desert mountain refugia. We used surface geology as a surrogate for ecological factors, based on its likely representation of edaphic boundaries and vegetation communities (Nano & Clarke, 2008; Perrigo et al., 2020; Rahbek et al. 2019b). We sought to determine: (a) to what extent geological substrate versus climatic factors predicts endemism, and (b) whether patterns of endemism are complimentary across guilds of taxa. First, we modelled the distributions of 82 endemic taxa across the three guilds of plants, land snails and vertebrates. We then combined the single taxa model outputs and modelled regional intraguild predictors of endemism. Finally, we examine interguild congruence in endemism and discuss the relevance of these results to developing strategies for conserving biodiversity in the region.

## 2 METHODS

### 2.1 Study area

**FIGURE 1:**
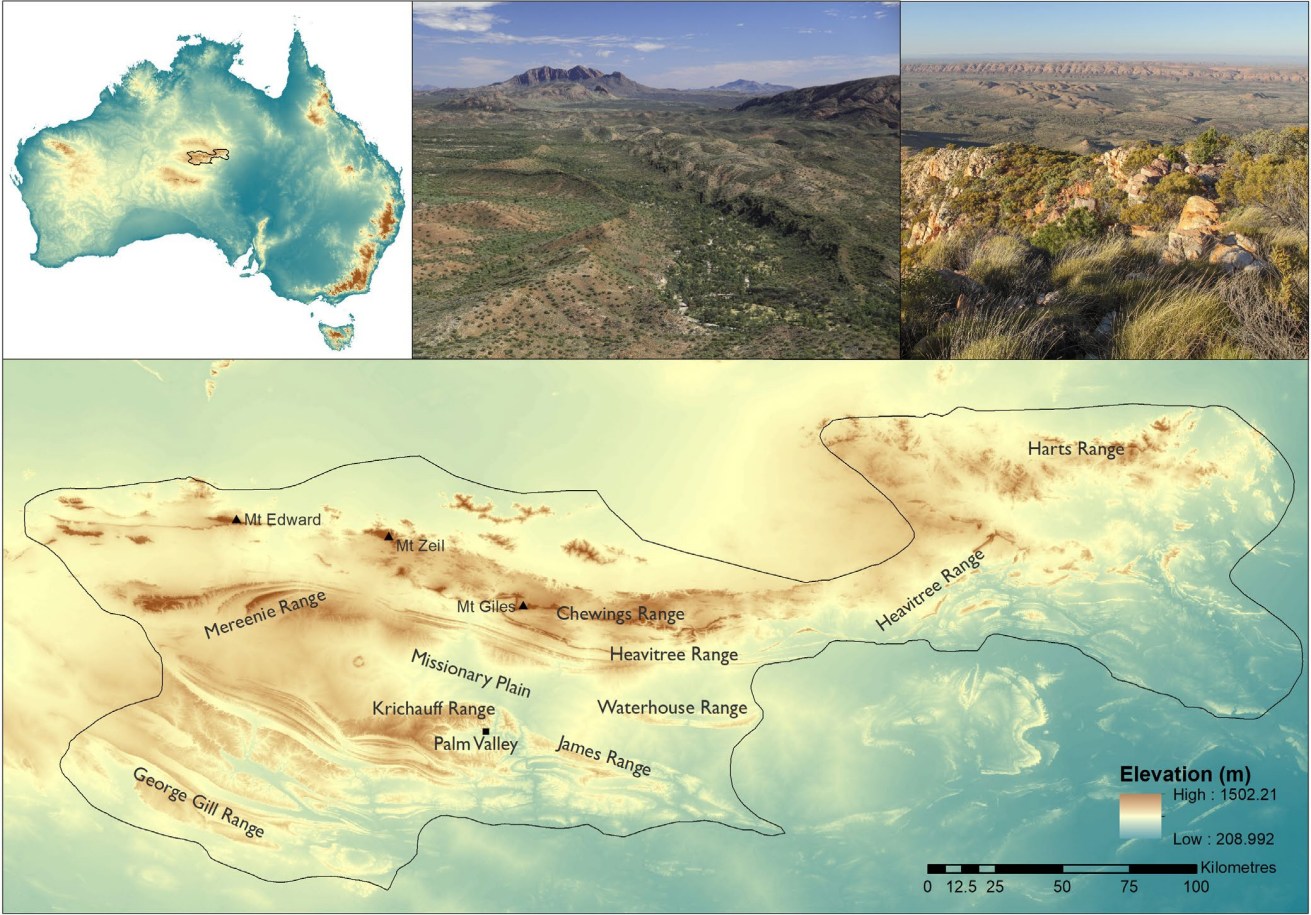
Elevation and landscape features of the study area, MacDonnell Ranges in the Australian Arid Zone (AAZ) (photos PJM). The region features diverse geologies and includes the granite Mt Zeil in the far north, quartzite Heavitree Range and Chewings Range in the central parts, and sandstone Krichauff Range and George Gill Range in the south

The MacDonnell Ranges is an area of elevated terrain (315–1531 m. a.s.l.) at the center of the AAZ that includes the highest mountains in Australia west of the Great Dividing Range (Figure 1). Here we focus on the MacDonnell Ranges bioregion and the neighboring Mt Chapple subregion, from the Burt Plain bioregion to the north, which together capture all broadly contiguous areas of complex terrain distinct from the neighboring flat sand deserts and plains (Thackway & Cresswell, 1995). The region uplifted during the “Alice Springs Orogeny” around 300–450 Ma (Shaw, 1991). Surface geology is diverse and comprises granites and gneisses in the north, east‐west running quartzite mountain ranges with diverse geologies (e.g., conglomerate, dolomitic limestone, gneiss, granite) in the intervening valleys, and sandstone ranges in the south (https://data.gov.au/dataset/ds‐dga‐48fe9c9d‐2f10‐49d2‐bd24‐ac546662c4ec/details). Broad vegetation types of the study area are frequently differentiated across distinct edaphic boundaries (Nano & Clarke, 2008) and include spinifex (*Trioda* spp.) grasslands, tall shrublands (*Acacia* spp.), alluvial woodlands and grasslands, and low chenopod shrublands (Perry & Lazarides, 1962). Climate of the region is semiarid with highly variable rainfall, cool winters, and hot summers. Predicted mean annual rainfall varies from 234–412 mm across the study area and is typically higher in the north, especially on the higher mountain ranges (BIOCLIM; Busby 1991). Predicted mean annual temperatures vary from 17–24°C with a negative relationship between elevation and temperature (BIOCLIM; Busby 1991). Cattle grazing is the dominant land use in the study area with Aboriginal land and protected areas comprising most of the remainder.

### 2.2 Single taxa species distribution models

We limited our analysis to endemic or near endemic taxa entirely or predominantly restricted to the study area. We defined the latter as taxa only known from the study area and a single outlier location and/or ≤5% of total records outside of study area. We sourced records of endemic plant species from the Australasian Virtual Herbarium (https://avh.ala.org.au/#tab_simpleSearch) and land snail and vertebrate records from the Atlas of Living Australia (https://www.ala.org.au/). We defined taxa as described species, nominal phrase name species (plants awaiting description), and evolutionarily significant units (ESU; Moritz et al., 2008) comprising genetically divergent populations (genetic data on ESU available for some vertebrates only; Oliver & McDonald, 2016). We screened for accuracy by removing records whose location field did not match known locations (e.g., a record in a valley with the location name assigned to a nearby mountain), and precision by removing records with less than four decimal places (decimal degrees).

For taxa with five or less location records (5 of 38 plant taxa; 3 of 27 snail taxa; 0 of 17 vertebrate taxa), we determined distribution using convex polygons (3–5 records) or by buffering records by 500 m (≤ 2 records). For taxa with more than five records, we ran species distribution models using Maxent (v. 3.4.1), with a standard set of climate and terrain variables likely to influence occurrence and the background extent set as the study area. Maxent is a machine‐learning presence‐only model that minimizes the relative entropy of estimated probability densities between taxa presences and the background landscape (Elith et al., 2011). Maxent often outperforms other SDM methods in predictive accuracy and is robust to small sample sizes (Hernandez et al., 2006; Wisz et al., 2008). We interpret our Maxent model outputs as indices of habitat suitability, thus forgoing the assumptions of sampling and probabilistic outputs (Merow et al., 2013), though we did account for spatial biases in taxa sampling (see below). If a taxa SDM predicted areas of high suitability well outside of the extent of location records, we interpreted this as taxa as being ecologically specialized (i.e., not at equilibrium with climate) and we added a geological substrate covariate to the model (see below). For the standard set of covariates, we screened for collinearity using Pearson's product‐moment correlation coefficient and removed one of pairs of highly correlated variables (*r* ≥ 0.7), resulting in nine covariates, including five climate variables from WorldClim (https://worldclim.org/data/bioclim.html): BIO01—annual mean temperature, BIO03—isothermality, BIO05—max temperature of warmest month, BIO12—annual rainfall, BIO17—dry quarter rainfall, and four terrain variables: elevation, aspect, slope, and terrain ruggedness index (TRI; Riley et al., 1999). The terrain variables were based on a 1 s digital elevation model (resolution c. 28 m) sourced from Geoscience Australia (https://www.ga.gov.au/). We resampled all climate variables to the terrain resolution using ArcMap (v. 10.7.1). For SDMs where we incorporated geology (i.e., habitat specialists), we used the Surface Geology of Australia 1:1 M dataset 2012 edition (https://data.gov.au/dataset/ds‐dga‐48fe9c9d‐2f10‐49d2‐bd24‐ac546662c4ec/details), wherein the study area data was compiled from 1:250,000 scale geology maps. Depending on the geographic spread of taxa records, we incorporated a categorical geology covariate representing either all areas of the same geology type or a discrete block(s) of a geology type. Because occurrence data typically has geographic bias (e.g., closer to roads), it is important to account for this variation in sampling effort in the modelling process (Kramer‐Schadt et al., 2013). We created separate bias layers for plants, land snails, reptiles and amphibians, mammals, and birds to reflect differences in sampling bias between these groups, by summing the total number of records of all species (including nonendemics) from each group within 5 km grid cells across the study area. The relevant bias layer was applied to each model and we applied the default Maxent settings (Merow et al., 2013). In addition to the standard cloglog suitability map outputs, we created thresholded (presence/absence) output maps based on maximum sensitivity plus specificity. All output maps were assessed for plausibility based on expert judgment of the authors.

### 2.3 Predictors of regional intra‐guild endemism

To create regional endemism maps we summed the individual taxa cloglog Maxent outputs for all taxa within each of the three guilds—plants, land snails, and vertebrates. We followed this approach, rather than thresholding outputs to presence/absence, to retain information on the gradient of suitability (a value of 0.5 is equivalent to a 50% probability of occurrence). We created a raster surface of mean endemism for each of the three guilds using a 5 × 5 km grid in ArcMap (v. 10.7.1). We then resampled all covariates used in the SDMs to the 5 km scale and added the new covariate “geo diversity”, determined as the number of unique geological surface types within each 5 km grid. We screened covariates for multicollinearity by regressing each covariate against all others and calculating variance‐inflation factors (VIF) in R (R Core Team, 2020) using the cars package (Fox et al. 2012), removing one of pairs of variables where the VIF was >4. We ran generalized additive models (GAMs) of endemic suitability with all remaining covariates in R (R Core Team, 2020) using the mgcv package (Wood, 2011), removing any covariates that had no significant relationship with endemism (*p* ≥ .05). Comparison of our GAM models to generalized linear models (GLMs) showed improved explained deviance (>10%) in all cases so we continued our analyses using GAMs. We checked smoothers using the gam.check() function, adjusting k until *p* ≥ .05, before inspecting qqplots and histograms to confirm that all model assumptions were met. We ran Morans I tests using the “ape” package (Paradis & Schliep, 2019) which revealed significant spatial autocorrelation in model residuals for all three endemism guilds. To account for this spatial autocorrelation, we incorporated a residual autocovariate term in each GAM (Crase et al., 2012). To assess the contribution of each covariate to the final GAMs, we recorded the drop in deviance explained when removed.

### 2.4 Hotspots and congruence in regional endemism

We determined regional hotpots of endemism based on the endemism raster surfaces (i.e., 5 km grid scale) for each guild using the Hot Spot Analysis (Getis‐Ord Gi*) in ArcMap (v. 10.7.1). We used the recommended default fixed distance band conceptualization of spatial relationships and the Euclidean distance method. From the hotspot output surfaces, we selected grids with a *p*‐value of <.01 to create our guild endemism hotspot maps and combined these to visualize cross‐guild overlap in endemism. Finally, to assess adequacy of protection for the endemism hotspots we calculated the percentage of hotspot cells that intersect the protected area network (World Protected Areas Database; https://www.protectedplanet.net/) within each guild and for the three guilds combined in ArcMap (v. 10.7.1).

## 3 RESULTS

### 3.1 Single taxa distribution models

**FIGURE 2:**
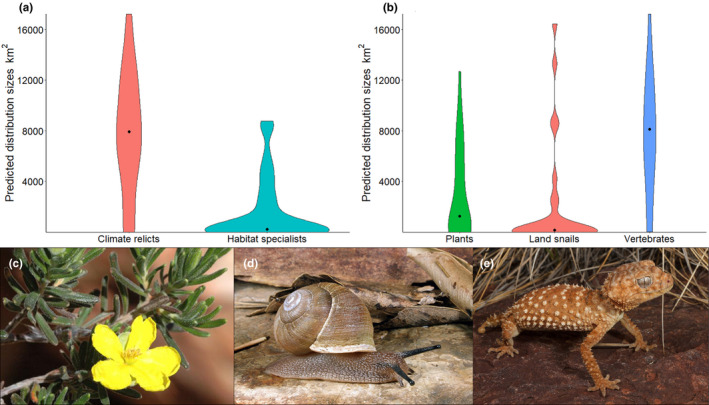
Violin plots of predicted geographic range sizes for: (a) climate relict (*n* = 28) and habitat specialist (*n* = 54) taxa, and (b) endemic plant (*n* = 38), land snail (*n* = 27) and vertebrate (*n* = 17) taxa (black dots represent median distribution sizes), and photographs of example taxa in each guild: (c) the plant *Hibbertia* sp. Chewings Range (Photo: Andrew Schubert, (d) the land snail *Sinumelon bednalli* (Photo: Vince Kessner), and (e) the vertebrate *Nephrurus amyae* (Photo: Chris Jolly), in the MacDonnell Ranges, Australian Arid Zone. Geographic range sizes were predicted from Maxent SDMs with the maximum training sensitivity plus specificity threshold

Twenty‐seven of the 38 (71%) endemic plant taxa, 23 of the 27 (85%) endemic land snail taxa, and four of the 17 (24%) endemic vertebrate taxa were assigned as habitat specialists (i.e., distributions were best explained by including geology in the SDMs; Appendix S1). Without the inclusion of geological surface covariates for these specialist taxa, the climate/terrain models predicted areas of high suitability well outside the extent of location records. Across all guilds, habitat specialists had significantly smaller geographic range sizes than climate relicts (Kruskal–Wallis chi‐squared = 24.69, *df* = 1, *p*‐value < .001) (Figure 2a). Larger percentages of habitat specialists corresponded to much narrower geographic range sizes for plants (median = 1,241.14 km^2^) and land snails (median = 147 km^2^), compared with vertebrates (median = 8,107 km^2^) (Figure 2b). There was an overall significant difference in geographic range sizes between guilds (Kruskal–Wallis chi‐squared = 15.496, *df* = 2, *p*‐value < .001) and a pairwise Wilcoxon test showed that vertebrate range size differed from plants (*p* = .001) and land snails (*p* = .001), but not between plants and land snails (*p* = .125).

Endemic plant taxa belong to 18 families, with Asteraceae (7 taxa), Fabaceae (5 taxa), and Poaceae (5 taxa) most represented. Some of the temperate families and autochthonous genera are represented by only one endemic species (e.g., *Leucopogon, Ricinocarpos* and *Actinotus*). The endemic land snail fauna was dominated by the Camaenidae and particularly *Catellotrachia* spp. (17 of 27 taxa). This radiation is restricted to the AAZ (MacDonnell Ranges and Central Ranges regions), characterized by species with short‐ranges and mostly allopatric distributions associated with discrete blocks of exposed rocky ranges with varying geological origins, and includes several threatened species. While most land snail taxa had geographic range sizes < 1,000 km^2^, four outlying taxa had predicted range sizes > 5,000 km^2^: *Granumelon adcockianum, G. grandituberculatum, Catellotrachia setigera,* and *Sinumelon expositum* (Figure 2b; Appendix S1). Fifteen of the 17 endemic vertebrate taxa were reptiles and seven of these were geckos (Gekkonidae). Most vertebrate taxa were associated with rocky substrates (evidenced by the importance of the TRI covariate) but occurred over large parts of the study area and on multiple geology types (Appendix S1). The four putative habitat‐specialist vertebrates that occurred on a narrower subset of geologies were all geckos: *Heteronotia fasciolatus; Oedura cincta*, *O. luritja,* and *Strophurus intermedius*.

### 3.2 Regional endemism

**FIGURE 3:**
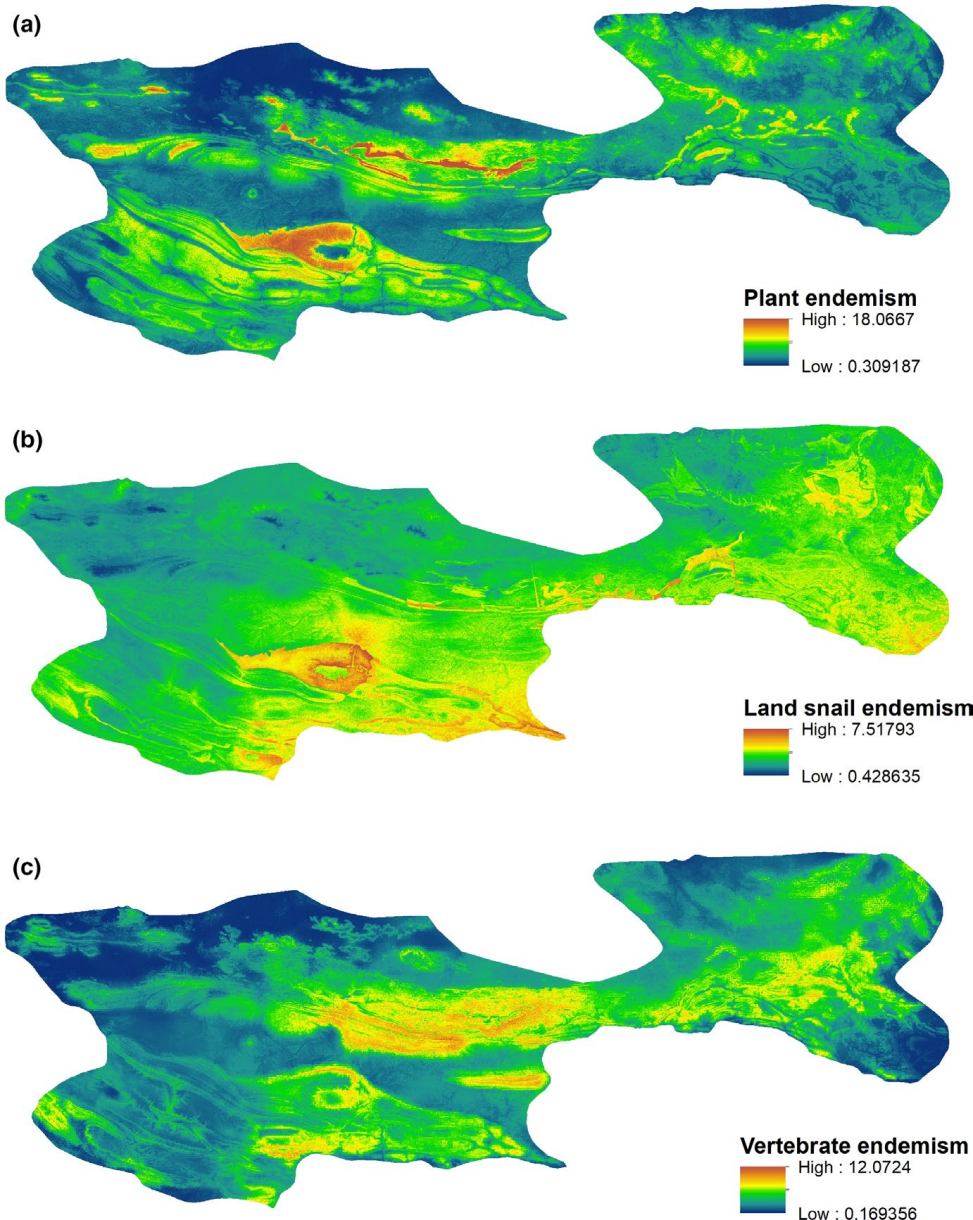
Endemic suitability for plant (a) (*n* = 38), (b) land snail (*n* = 27) and (c) vertebrate (*n* = 17) taxa in the MacDonnell Ranges, Australian Arid Zone. Suitability calculated by summing Maxent SDM model outputs (cloglog) for all endemic taxa in each biotic group

**FIGURE 4:**
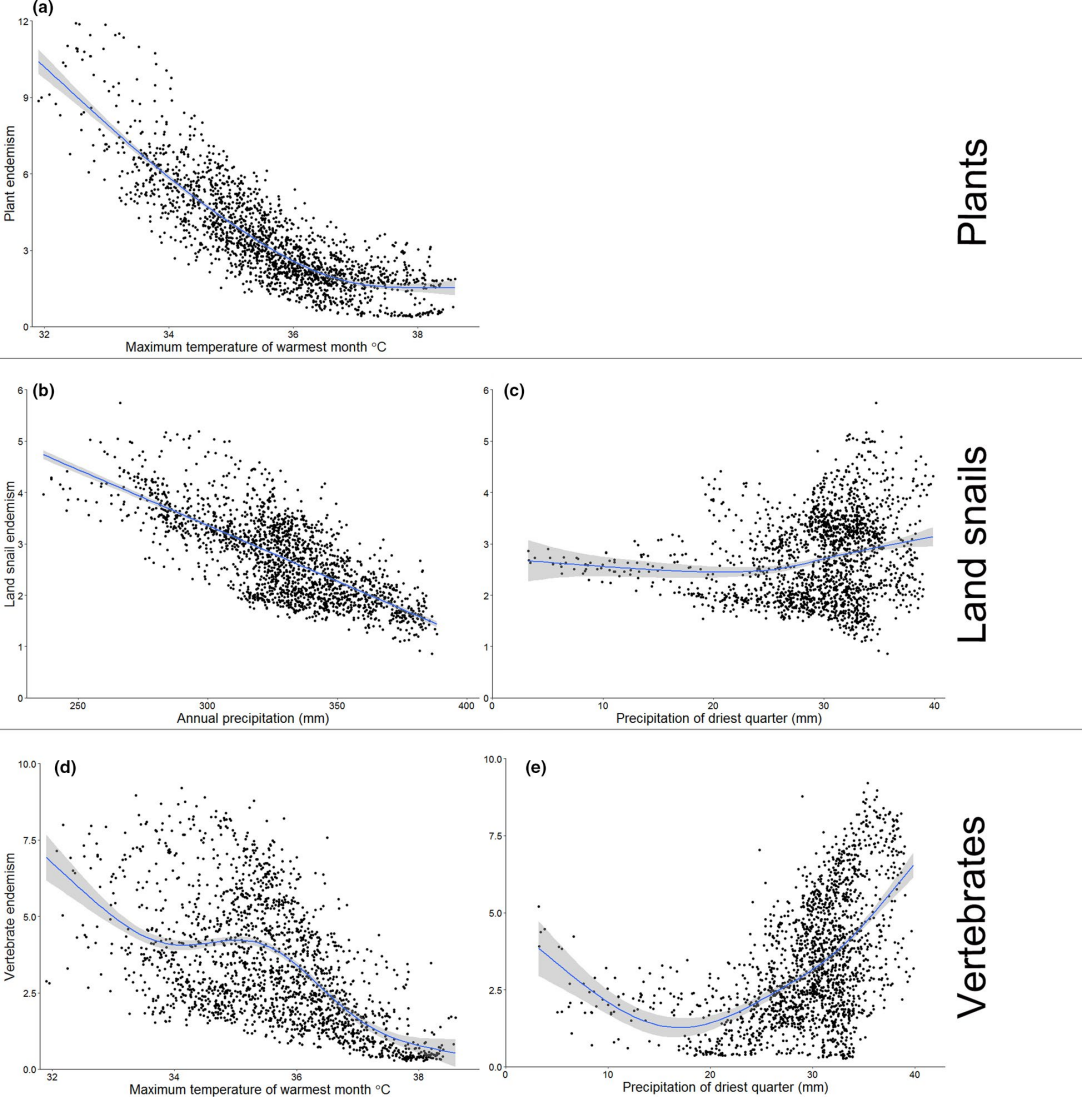
Relationships between regional endemism and important covariates (>10% contribution explained variance in full GAM models) for (a) plants, (b–c) land snails, and (d–e) vertebrates in the MacDonnell Ranges, Australian Arid Zone. Fitted Generalised Additive Model smoother relationships with 95% confidence intervals

Summed taxa SDMs revealed contrasting patterns of regional endemism between the three guilds (Figure 3) and that climate variables were the most important predictors of endemism in all guilds (Figure 4; Table 1). While geology variables dominated in many individual land snail and plant taxa SDMs (Appendix S1), they contributed little to the overall regional endemism models (Figure 4; Table 1). Plants had the highest maximum endemism value and the most discrete areas with high endemism values (Figure 3a). The most important predictor of plant endemism was maximum temperature of warmest month, with a negative relationship between temperature and endemism (Figure 4a; Table 1). Of the three guilds, land snails had the lowest maximum endemism value, suggesting higher levels of endemism turnover for this guild (Figure 3b). There was a negative relationship between annual precipitation and land snail endemism (Figure 4b) and a weaker but positive relationship between dry quarter rainfall and land snail endemism (Figure 4c). Maximum vertebrate endemism was high and vertebrate endemism was more evenly distributed, compared with plants (Figure 3c). This pattern indicates that many endemic vertebrates broadly co‐occur across significant portions of the study region. There was a negative and weakly hump‐shaped relationship between maximum temperature of warmest month and vertebrate endemism (Figure 4d) and a positive relationship between dry quarter rainfall and vertebrate endemism (Figure 4e).

**TABLE 1:** Residuals autocovariate (RAC) generalized additive models (GAMs) explaining endemism for plants, land snails and vertebrates in the MacDonnell Ranges, Australian arid zone

Model (% deviance explained)	Covariates*	edf (estimate degrees of freedom)	*F*‐test	% drop in deviance explained with covariate removed
Plants (97.0%)	s(TRI)	13.262	60.29	1.3%
	s(BIO05)	3.959	5,839.52	36.8%
	s(BIO12)	3.941	723.50	4.6%
	s(BIO17)	3.954	515.69	3.2%
	s(RAC)	3.444	1923.94	–
Land snails (96.1%)	s(TRI)	8.716	8.149	0.2%
	s(Slope)	3.925	323.586	2.8%
	S(Elevation)	3.933	998.434	8.7%
	s(BIO12)	8.574	1,066.167	20.6%
	s(BIO17)	8.192	597.959	11.4%
	s(RAC)	8.525	811.192	–
Vertebrates (95.4%)	s(TRI)	13.952	82.62	2.9%
	s(Geo_div)	1.000	62.50	0.2%
	s(BIO05)	3.992	1,118.98	11.1%
	s(BIO12)	3.943	995.04	9.8%
	s(BIO17)	3.844	11,969.34	19.5%
	s(RAC)	3.469	2056.45	–

Abbreviations: BIO05, maximum temperature of warmest month; BIO12, annual precipitation; BIO17, dry quarter precipitation; Geo_div, geological surface diversity; RAC, residuals autocovariate; TRI, terrain ruggedness index.

* *p* values for all covariates < .001.

**FIGURE 5:**
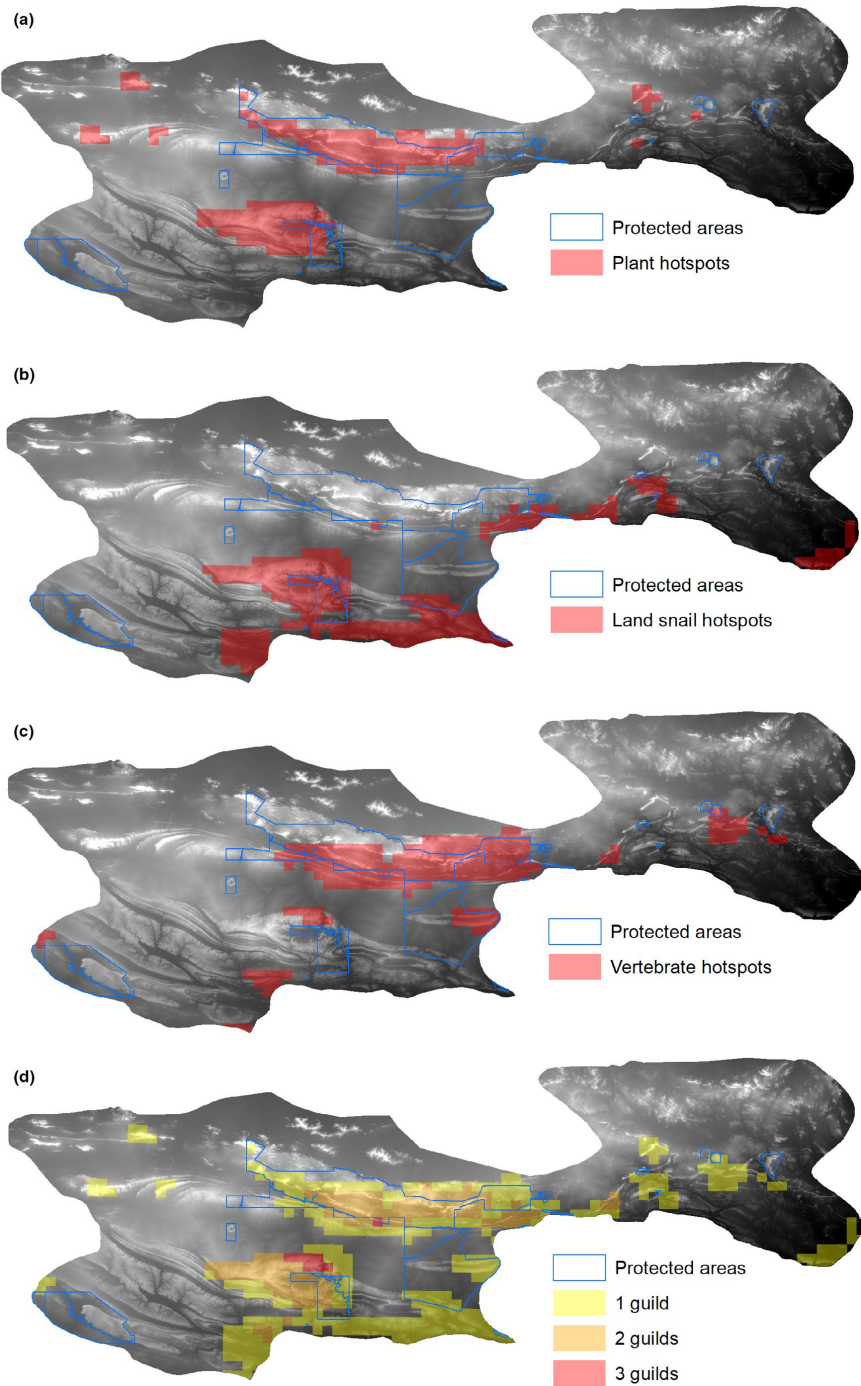
Predicted endemism hotspots for (a) plants, (b) land snails, (c) vertebrates, and (d) all three guilds combined in the MacDonnell Ranges, Australian Arid Zone. Hotspots determined using Getis‐Ord Gi in ArcGIS and 5 km grids selected at *p* = .01

Plant endemism peaked on the quartzite Chewing Range and Heavitree Range, and associated outliers, in the north‐west, and on the sandstone Krichauff Range north and west of Palm Valley (Figures 3a and 5a). The highest land snail endemism values were on the sandstone Krichauff Range and James Range in the central‐south and south‐east, and along the central‐east section of the quartzite Heavitree Range in the north (Figures 3b and 5b). Vertebrate endemism peaked across various geologies in the central and eastern parts of the study area (Figures 3c and 5c).

There was low congruence in endemism hotspots between the three guilds (Figure 5). Guild hotspots were poor predictors of hotspots in other guilds: plant hotspots predicted 24% of land snail and 38% of vertebrate hotspots, snail hotspots predicted 31% of plant and 14% of vertebrate hotspots, and vertebrate hotspots predicted 40% of plant and 12% of land snail hotspots. Guild hotspots were poorly to moderately captured in the regional protected area network: 52% of plant hotspots, 28% of snail hotspots, and 57% of vertebrate hotspots intersected protected areas. The only substantial area of predicted hotspot for all three guilds lies outside the protected area network, immediately north of Finke Gorge National Park (Figure 5d).

## 4 DISCUSSION

While considerable work been invested in documenting and understanding patterns and drivers of endemism in temperate, and especially tropical, mountains (Rahbek et al. 2019a), the biota of arid‐zone mountains have been relatively overlooked. Our analysis of endemism in a geologically ancient and low elevation mountain system in Australia's arid center highlights contrasting distributional patterns across plants, land snails, and vertebrates. Localized ecological factors (namely geological substrate) predict endemism to varying degrees across these three guilds, however across all taxa there is a strong signature that cooler climate and higher precipitation in mountain areas have played an important role in the persistence of relictual taxa in this arid system.

### 4.1 Patterns and predictors of endemism in an arid mountain refugium

Rahbek, Borregaard, Colwell, et al. (2019) highlighted the diverse array of processes by which mountains may generate and maintain exceptionally diverse biotas, including many locally endemic species. While our study focused on contemporary predictors of endemism, it nonetheless highlighted that a suite of similar climatic and ecological processes underpin endemism in an arid zone mountain range. Specifically, many individual plant and land snail taxa endemic to the MacDonnell Ranges region were tightly linked with geology, corresponding to small distributions, and likely driven by ecological specialization and physical barriers to dispersal, respectively. Conversely, most endemic vertebrate taxa were not constrained to specific geological substrates and occurred over larger areas, probably reflecting greater mobility and more ecological generalization. However, across all guilds of taxa higher rainfall and cooler temperatures were significant predictors of diversity for endemic taxa, a pattern mirroring results from tropical mountain ranges across the world (Rahbek et al. 2019a).

Of the three guilds we investigated, plants are the group for which edaphic factors are often the best predictor of endemism (Corlett & Tomlinson, 2020). Therefore, we expected that plant endemism in the MacDonnell Ranges would also be closely correlated with geology and terrain. Consistent with this prediction, we found a number of topographic specialists (e.g., *Macrozamia macdonnellii*) which occur widely across the study area that are associated with microclimates that may provide a buffer from desiccation (Fitzsimons & Michael, 2017; Preece et al., 2007). Further endemic plant taxa are associated with rare geology types (e.g., deeply weathered tertiary deposits for *Olearia macdonnellensis,* gravelly ranges with acidic–neutral soils for *Scaevola* sp. Mt Liebig) and may be edaphic specialists (Corlett & Tomlinson, 2020). However, at the regional scale there was also a strong negative relationship between maximum summer temperatures and plant endemism. Most notably, hotspots of plant endemism occur on the sections of quartzite, sandstone and granite mountain ranges with the highest elevations and elevational ranges in the study area. These areas may have functioned as refugia by enabling upslope movements during periods of warming (Lenoir et al. 2008; Walther et al., 2005) and now support distinctive high elevation plant communities (Nano et al., 2019). Some of these high‐elevation taxa occur on both quartzite and sandstone (e.g., *Hakea grammatophylla, Hibbertia* sp. Chewings Range), reinforcing the idea of climate limitation rather than ecological specialization per se. Several endemic plants are the only representatives of their genera in the AAZ or from the central parts of the arid zone (e.g., *Actinotus, Amperea, Caesia, Leucopogon, Macrozamia, Ricinocarpos*). One high‐elevation taxon diverged from its nearest relative in a temperate biome coincident with mid‐Miocene aridification (*Hakea grammatophylla*; Cardillo et al., 2017), further supporting the hypothesis of the MacDonnell Ranges as a climate refugia (Byrne et al., 2008). Overall, the combination of contemporary restriction to high elevations and highly disjunct distributions supports the hypothesis that the cooler and wetter, relatively high elevation mountain ranges with substantial elevational ranges have been critical to the persistence of many endemic plant taxa.

The endemic land snail fauna of the MacDonnell Ranges is unique among the three guilds in having relatively low levels of accumulated endemism. Specifically, land snail endemism hotspots supported fewer species than plant and vertebrate hotspots. This pattern was mostly driven by the dominant land snail genus *Catellotrachia*, which is characterized by many short‐range taxa with mostly allopatric distributions (e.g., numerous taxa only known from a single gorge or section of mountain range; Solem, 1993; Woinarski, 2007). With their small size and rock‐sealing aestivation requirements (Solem, 1993), relatively minor geographic boundaries may have promoted allopatric speciation in *Catellotrachia* during periods of aridification. While this hypothesis awaits phylogenetic testing for *Catellotrachia*, populations of another endemic genus, *Granulomelon*, diverged during severe aridification in the mid‐Pleistocene, with levels of population connectivity linked to differing aestivation strategies (rock‐sealing versus free‐sealing) (Criscione & Köhler, 2016). The negative relationship between annual rainfall and land snail endemism was surprizing given that these snails are only active after rain (Solem, 1993), however there was also a weaker positive relationship with dry quarter rainfall suggesting rainfall seasonality may be important. Further, the ability of land snails to aestivate for months or years without significant rainfall (Solem, 1993) presumably confers some ability to decouple from regional climatic patterns, explaining the important primary role of geology and dispersal barriers in explaining present‐day land snail distributions. Snails also provide the only evidence of endemic diversification within this region, further highlighting the attenuated and likely relictual nature of endemism in some other components of the biota.

Most endemic vertebrate taxa had relatively large geographic range sizes, occurred over many geological substrate types, and broadly co‐occurred with other endemic vertebrates over substantial portions of the study area. These observations of broadly occurring vertebrate taxa are consistent with the hypothesis that more short‐range taxa were largely unable to persist through climate instability in the region (e.g., Pleistocene climate oscillations; Byrne et al., 2008; Crisp et al., 2001), and contrasts with coastal arid mountain ranges in northern Africa and Australia that support suites of short‐range vertebrate taxa and high levels of intraspecific diversity (Doughty et al., 2016; Garcia‐Porta et al., 2017; Oliver & Doughty, 2016; Pepper et al., 2011, 2013). Compared to some of these other arid zone mountain ranges, the MacDonnell Ranges are also relatively small in areal extent and low in elevation, minimizing opportunities for allopatric divergence or segregation along elevational gradients. This, combined with the long history of aridification and absence of ameliorating coastal affects in the MacDonnell Ranges, may have favored taxa with some ability to be able to track climate shifts across diverse geologies and landforms.

The apparent relationships between climatic variables and vertebrate endemism may be linked to climate limitation. The negative relationship between maximum temperature and vertebrate endemism, and the positive relationship between endemism and dry quarter rainfall, are consistent with the climate refugia hypothesis for the MacDonnell Ranges. Dated phylogenies have also revealed vertebrate divergences coinciding with periods of aridification in the Miocene and Plio‐Pleistocene (Byrne et al., 2008; Oliver & McDonald, 2016). The peak of endemism at mid temperatures suggests that the reptile‐dominated endemic vertebrate faunas also are constrained by cooler temperatures in the highest elevation parts of the region (McCain, 2006; Tallowin et al., 2017). This again suggests that vertebrate endemism in these arid ranges is linked to many of the same climatic drivers as more mesic systems (Rahbek et al. 2019a, 2019b).

### 4.2 Conserving endemism in an arid mountain refugium

Identifying hotspots of evolutionary biodiversity or endemism is a key strategy for conservation planning and management (Rosauer et al., 2016). However, the distribution and structuring of endemic species within broad hotspots can vary greatly across taxa (Moritz et al., 2001). In this study, the divergent patterns of endemism shown across the three focal guilds, highlight how a range of conservation strategies are required to conserve endemism in the MacDonnell Ranges, and potentially other arid‐zone mountain biotas.

Our hotspot analyses identified significant intraregional refugia (e.g., refugia within refugia; Gómez & Lunt, 2007) that are likely to become increasingly important with global warming and should be foci for land management addressing a range of threats (e.g., wildfire, invasive species). The Krichauff Range hotspot for all three guilds lies outside of the protected area network. This hotspot contains an active oil and gas field and, while this operation has a relatively small footprint of wells and unsealed roads, the potential for long‐term impacts on this refugia must be considered in any proposed expansions or rehabilitation. The Krichauff Range and western quartzite ranges plant hotspots (including Mt Edward) are on Aboriginal (indigenous Australian) freehold land and, given their status as significant refugia and threatened species refuges (Pavey et al., 2017), together with the presence of active Indigenous ranger groups in both areas, they are strong candidates for Indigenous protected areas if supported by traditional owners (Smyth, 2006). Land snail hotspots supported fewer species than the other guilds and targeted management of individual short‐range taxa may be required to protect them from invasive grasses (e.g., *Cenchrus ciliaris*) and wildfire (Woinarski, 2007). In contrast, because the vertebrate taxa have large geographic range sizes and are climate relicts (with the excepton of two threatened species; McDonald et al., 2018; Pavey et al., 2010), they are likely to be less at risk from localized habitat disturbance than the other guilds. However, the signal of climate limitation suggests it will be useful to track any changes in distribution patterns associated with global warming, with analysis of atlas data, including citizen‐collected records, providing a cost effective approach for this conspicuous and well‐sampled guild (Mcdonald et al., 2015), complementing strategic monitoring of priority species.

Of particular conservation concern is the suite of six plant species restricted or mostly restricted to the highest parts of the quartzite mountains in the central‐west parts of the study area. These short‐range species appear to occur at the upper limits of their thermal niche and will have limited capacity to shift upslope in response to the ongoing trend of increasing maximum temperatures in the study region (Appendix S2). None of these species are currently being monitored for phenological or distribution changes (e.g., Guerin et al., 2012) and only one is listed as threatened under national legislation (*Prostanthera schultzii*). While increasing temperatures may be the dominant threat for the high‐elevation flora, managing other stresses such as wildfire through management burning, and weeds through restricting access of human vectors (Clarke et al., 2005; Pickering & Mount, 2010; Woinarski, 2007), could improve the resilience of these high elevation‐specialists given that synergistic processes frequently drive extinction events (Brook et al., 2008).

## 5 CONCLUSIONS

We found an overarching signal that, even in a low elevation and relatively low rainfall arid zone mountain range, climatic gradients still play a dominant role in the persistence of endemic taxa. This mirrors studies in other mountain systems in different biomes which also indicate that climate is the dominant correlate of endemism (Rahbek et al. 2019a). Nonetheless individual taxa and especially different guilds of taxa still show contrasting patterns at local scales, highlighting the need to couple comprehensive regional planning for the protection of critical climate refugia with conservation strategies targeting at‐risk taxa and hotspots. The strong correlation between higher rainfall and/or cooler temperatures with endemism across all guilds suggests observed trends over the last 50 years towards hotter and drier climates will stress montane areas in much the same was as they threaten upland biotas in other biomes, with short‐range endemic high‐elevation taxa being particularly vulnerable.

## CONFLICT OF INTEREST

None declared.

## AUTHOR CONTRIBUTIONS

**Peter J. McDonald:** Conceptualization (lead); data curation (lead); formal analysis (lead); methodology (lead); visualization (lead); writing–original draft (lead); writing–review and editing (lead). **Peter Jobson:** Data curation (supporting); methodology (supporting); writing–review and editing (supporting). **Frank Köhler:** Data curation (supporting); methodology (supporting); writing–review and editing (supporting). **Catherine E. M. Nano:** Data curation (supporting); methodology (supporting); writing–review and editing (supporting). **Paul M. Oliver:** Conceptualization (supporting); data curation (supporting); methodology (supporting); writing–original draft (supporting); writing–review and editing (supporting).

## Supporting information

Appendix S1

Appendix S2

## Data Availability

The data are available in the Dryad database under the following link: https://doi.org/10.5061/dryad.b5mkkwhcg.
