# Supplementary material for: The living heart: Climate gradients predict desert mountain endemism
Source: Ecol Evol. 2021 Mar 15;11(9):4366–78. doi: 10.1002/ece3.7333 (PMC8093673; doi:10.1002/ece3.7333)
Supplement: Supplementary file 2 — Appendix S2 [file ECE3-11-4366-s002.docx]

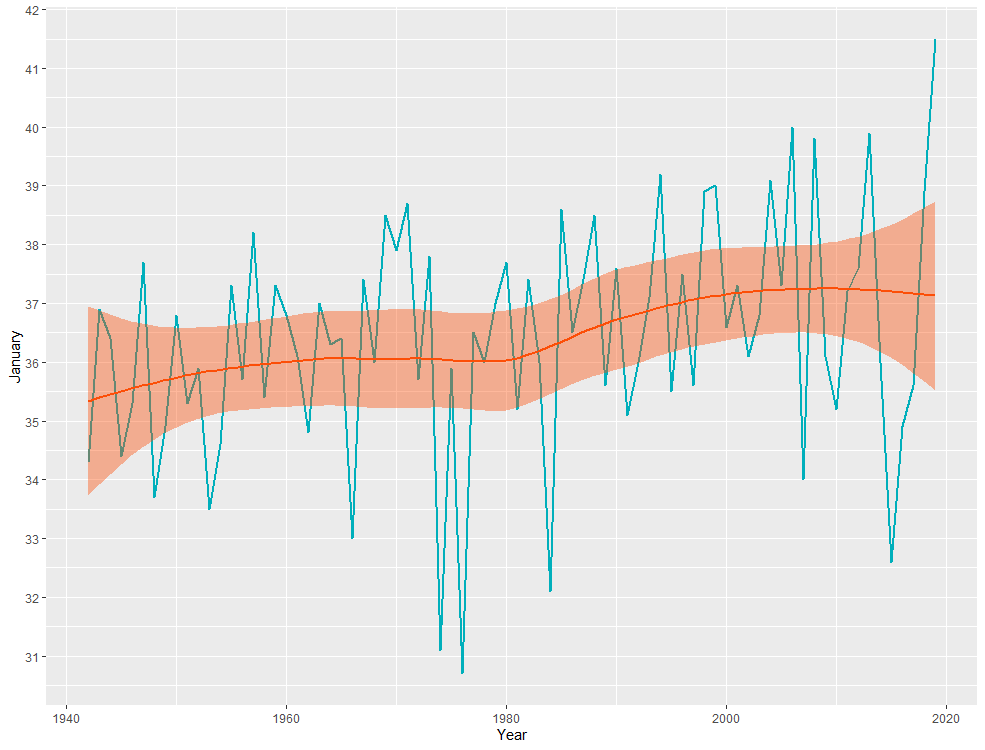


Figure S2. Mean January maximum temperatures (°C) at Alice Springs airport between 1942-2019, including mean trendline and 95% confidence intervals (<http://www.bom.gov.au/>).
